# Mobile Health Insurance System and Associated Costs: A Cross-Sectional Survey of Primary Health Centers in Abuja, Nigeria

**DOI:** 10.2196/mhealth.4342

**Published:** 2016-05-17

**Authors:** Emeka Chukwu, Lalit Garg, Godson Eze

**Affiliations:** ^1^ University of Liverpool Faculty of Computer Science and Engineering Liverpool United Kingdom; ^2^ University of Malta Faculty of Information and Communication Technology Msida Malta; ^3^ Delta State University Teaching hospital (DELSUTH) Department of Community Medicine Oghara Delta State Nigeria

**Keywords:** mobile health, mHealth, eHealth, health financing, health insurance, public health informatics

## Abstract

**Background:**

Nigeria contributes only 2% to the world’s population, accounts for 10% of the global maternal death burden. Health care at primary health centers, the lowest level of public health care, is far below optimal in quality and grossly inadequate in coverage. Private primary health facilities attempt to fill this gap but at additional costs to the client. More than 65% Nigerians still pay out of pocket for health services. Meanwhile, the use of mobile phones and related services has risen geometrically in recent years in Nigeria, and their adoption into health care is an enterprise worth exploring.

**Objective:**

The purpose of this study was to document costs associated with a mobile technology–supported, community-based health insurance scheme.

**Methods:**

This analytic cross-sectional survey used a hybrid of mixed methods stakeholder interviews coupled with prototype throw-away software development to gather data from 50 public primary health facilities and 50 private primary care centers in Abuja, Nigeria. Data gathered documents costs relevant for a reliable and sustainable mobile-supported health insurance system. Clients and health workers were interviewed using structured questionnaires on services provided and cost of those services. Trained interviewers conducted the structured interviews, and 1 client and 1 health worker were interviewed per health facility. Clinic expenditure was analyzed to include personnel, fixed equipment, medical consumables, and operation costs. Key informant interviews included a midmanagement staff of a health-management organization, an officer-level staff member of a mobile network operator, and a mobile money agent.

**Results:**

All the 200 respondents indicated willingness to use the proposed system. Differences in the cost of services between public and private facilities were analyzed at 95% confidence level (*P*<.001). This indicates that average out-of-pocket cost of services at private health care facilities is significantly higher than at public primary health care facilities. Key informant interviews with a health management organizations and a telecom operator revealed high investment interests. Cost documentation analysis of income versus expenditure for the major maternal and child health service areas—antenatal care, routine immunization, and birth attendance for 1 year—showed that primary health facilities would still profit if technology-supported, health insurance schemes were adopted.

**Conclusions:**

This study demonstrates a case for the implementation of enrolment, encounter management, treatment verification, claims management and reimbursement using mobile technology for health insurance in Abuja, Nigeria. Available data show that the introduction of an electronic job aid improved efficiency. Although it is difficult to make a concrete statement on profitability of this venture but the interest of the health maintenance organizations and telecom experts in this endeavor provides a positive lead.

## Introduction

### Background

This study aims to document costs associated with and provide justification for adoption of mobile phone as an alternative to drive the uptake of community-based health insurance schemes (CBHIs). Studies have shown that one of the biggest challenges of health care systems in developing countries is financing [1]. Data from World Bank puts the percentage of persons living on less than a dollar a day in Nigeria at 68.0% [2]. The Presidential Task Force on maternal health in Nigeria, in a randomized research, found that 30% of participating pregnant women could not use maternal health services owing to their inability to pay [3]. The government remains the single major financier of health systems in Nigeria. Nigeria has slightly more than 21,808 public primary health centers (PHCs) as compared to 8290 private PHCs [4]. These numbers exclude the secondary and tertiary health facilities.

On the basis of current health insurance coverage estimates, most clients settle payments for health services out of pocket at point-of-service [5]. “Evidence from other developing countries have shown that catastrophic health spending can push people into poverty” [6]. Reasons for poor health insurance enrolment despite its potential for risk pooling has been the subject of much research [7-9]. Reimbursement methods adopted have equally been a source of debate [9]. Because capitation considers the volume of clients serviced for a period, the clients might not get optimum quality of care, thus adversely reducing patient health and satisfaction and subsequently, health outcome [10]. Other schools of thought dictate that the fee-for-service method has resulted in “overtreatment,” with multiple patient visits required for services [9]. Business experts on the other hand are looking for a “sweet spot” in this dilemma [11]. This may be the reason why Nigeria’s health insurance regulator, the National Health Insurance Scheme (NHIS), allows for a use of a hybrid of the two [5].

### Nigeria’s Health Insurance Adoption

The NHIS was setup to provide a regulatory framework to support the social health insurance system for Nigerians [5]. The NHIS puts the current insurance adopters at less than 4 million of the country’s total population of about 170 million [5]. This is less than 3% of the country’s population. This is quite poor when compared to that of Ghana, with a total population of 25 million and more than 14 million persons enrolled in 3 years [12]. These statistics indicate that the NHIS fell short of its target of 30% coverage by the end of 2015 as mandated by the Nigerian president [5]. The scheme recently issued a guideline for CBHI [13], proposing community prepayment and a not-for-profit model as the preferred method. States of the federation have also been authorized to run state-based health insurance schemes. Many communities and states currently do not possess the capacity to sustainably manage and track the complexity of an insurance scheme [8]. As a result, adoption has been very low, and the few CBHI pilots have not scaled. A scalable CBHI needs to solve the problem of premium tracking, management, and accountability. And technology has been shown to improve accountability and transparency [14]. If properly applied, technology has the potential to solve many of the thorny issues bedeviling the CBHI’s adoption at scale.

For the purpose of this study, all discussions referencing health insurance will refer to the CBHI hybrid focusing on maternal and child health. Various models of this scheme exist, one type, taking its operational model from its name, is organized and managed by community members through a committee sometimes called “Ward Development Committee”[15]. The committee manages drug purchases and other health facility spending. The premium is set by the community and maintained by committee members. It is also the responsibility of the committee to keep track of claims and spending and report back to the general community meetings [5,16,17]. This scheme has been piloted at different times in Lagos, Jigawa, Anambra, and Abuja. Some regions struggle to continue implementing the scheme after the initial pilot, whereas others stopped at the end of the pilot phase [7,17]. The current costing model covers health maintenance organization (HMO) administrative costs, NHIS administrative costs, capitation fees for the PHCs, and fee-for-service fees for secondary and tertiary health facilities [5]. The guidelines further stipulate that communities seeking to join the CBHI scheme should have more than 1000 enrollees or more than 50% of its population ready to enroll. NHIS has not fixed premium and reimbursement rates for CBHI. However, a pricing template is provided to guide the HMOs and the health facilities in reaching an agreement.

### Primary Health Centers in Abuja

Maternal and child health domain of PHCs in Abuja, Nigeria, was the scope of this work. Treatment received at a referral center was excluded to reduce complexity.

Abuja is the federal capital territory (FCT) of Nigeria. Its population is 2.29 million [4]. The health care structure in Nigeria is such that the federal government is responsible for the tertiary health facilities through the federal ministry of health and the state governments for the secondary health facilities through their hospital management boards, while the PHCs are the responsibility of the local government areas (LGAs). The directory of health facilities in Nigeria puts the number of PHCs in FCT at 559, and only 179 (32%) of these are publicly owned [4]. Abuja is administratively grouped into 6 area councils, equivalent of LGAs in the states of the Nigerian federation. Area councils in Abuja ideally manage these 179 public-owned PHCs. They provide operational and logistics support. The staff salaries of these PHCs are also the responsibility of the area councils [3].

### Digital Health

There has been a massive growth in and adoption of mobile phone and related services in Nigeria over the last decade [18]. Data from Nigerian Communications Commission show that Nigeria is one of the fastest growing markets for mobile telephony, with penetration near 90% and over 121 million active GSM lines as of September 2013 from 240,000 lines at inception in 2001 [18,19]. The growth of this technology has given rise to various uses such as car tracking, remote home surveillance systems, and many more, and this has since been extended to health care. This presents an opportunity to reach a larger proportion of the underserved with mobile technology-supported health services, particularly for insurance uptake. Many have already adopted this for health information and education through short message service (SMS) text reminders, and there is overwhelming evidence to show that adherence to drugs and hospital attendance improved with SMS text message reminders [20]. Although there is limited evidence on how the use of mobile technology beyond SMS can be linked to health outcomes [21], electronic medical records have been shown to reduce costs and errors [22]. Kumar and Bauer [22] also argued that it is possible to mine treatment data and other information from a properly implemented electronic health record system. Nigeria recognized the potential technology has to meet the ambitious Millennium Development Goals 4, 5, and 6. This culminated in the launch of the Saving of One Million Lives initiative [23]. At the launch of the initiative in December 2012, the government announced a partnership with mHealth Alliance to use information communication technology to support this initiative [24]. Kai-Lik Foh, Mobile Health Manager at the Association of GSM telecommunications operators suggested the application of mobile technology for health insurance in his 2011 article [25]. He listed the various stakeholders: payers, providers, purchasers, producers, and so forth. However, 3 years after this work, to our knowledge, no single paper on an operational or prototype system addressing this exists in the public domain.

## Methods

### Ethical Approval

This research was the output of the principal investigator’s masters level dissertation studies. Data collection and analysis were conducted between December 2013 and February 2014. The Research Ethics Committee of the University of Liverpool, United Kingdom, approved this study.

### Design

**Figure 1:**
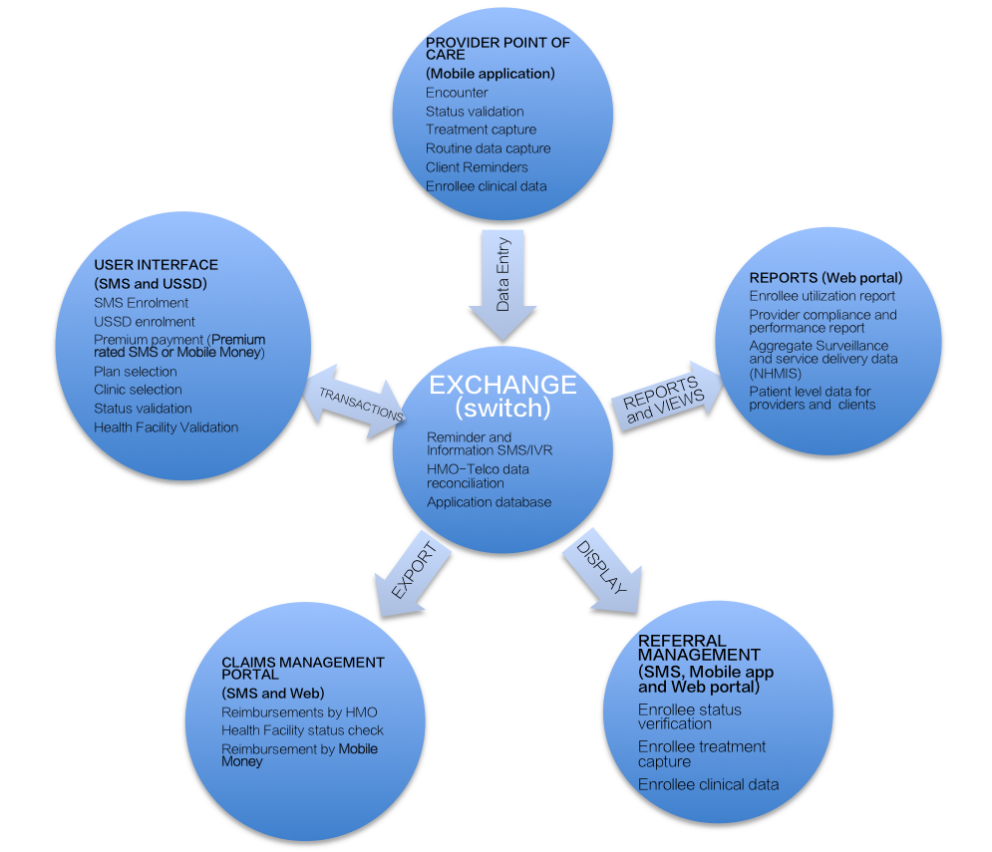
Prototype Software Conceptual Framework.

This study is an analytic cross-sectional survey, which used a hybrid of mixed methods, involving key informant interviews supported by a “throw-away’ prototype software demonstration. A review of the current literature was conducted to guide the appropriateness of the approach. Health facility clients and health care providers were interviewed using structured questionnaires. The income and expenditure at both the private and public PHCs were analyzed. A client and a provider were interviewed in each health facility visited. Fifty public and 50 privately owned PHCs were considered representative of the study area. A throw-away prototype software system was designed to guide respondents’ understanding of the electronic enrolment and encounter systems. The prototype software conceptual framework is shown in Figure 1. The software structure shows a combination of 5 different subsystems with special focus on end-user requirements. At the time of interview, only the “CommCare mobile point of care” was functional in this multifunctional system. This was sufficient for demonstration of the software to the health workers for interview purposes. The software setup and running costs were extrapolated using the software development life cycle methodology. Similarly, the out-of-pocket costs paid for health services were also documented to assess the current income of the PHC. The willingness to pay result was used to extrapolate proposed income per health facility. An assumption in the income extrapolation was that the outpatient visits would record 50 visits for various consultations per month.

### Recruitment

Two structured questionnaires were used for health facility clients’ (n=100) and the health workers’ (n=100) interviews. One hundred PHCs were randomly selected and visited by the research assistants. Fifty each of private and public primary health facilities were purposefully sampled to provide representative spread across public and private PHCs. The participant information sheet and the informed consent forms were read to the clients/health workers and health workers, and the consenting clients were interviewed after appending their signatures. One client and one health care provider were interviewed in each PHC visited. Interviewed clients had to be aged 18 years or older and must have received antenatal, immunization, or delivery service in the health facility visited. Client selection was on the first contact basis. Clients not meeting these criteria were excluded from the study, and the next available client was assessed and interviewed. The health worker interviewed was the most senior officer in the health facility at the time of visit by research interviewers. For confidentiality, all identifiable information was excluded from the questionnaire; to ensure data validity and reduce bias, research assistants and interviewers were enlightened on the objectives of the research and trained on how to ask and obtain answers for each of the questions to reduce bias and interobserver variation, as they were not supervised during the interviews. The health facility and LGA codes were adapted from the directory of the health facility [4].

The client questionnaire had 26 questions, which were grouped under 3 parts: A, B, and C. Part A of the client questionnaire contained questions that related to the reliability of the services in the health facilities. Information on the “time of last visit” was used to measure the frequency of health facility visits. Although the frequency of client falling ill and other factors might be beyond the scope of this research, these data were necessary to document costs necessary for good return on investment of any system targeting the primary health facility. Client’s perception of the service provided was captured using the “service rating” question. This was also validated using the “willingness to recommend others to the health facility,” question, as it is expected that a client would only be willing to recommend others to the service if it was above average by their rating. This might not necessarily be an accurate measure of quality, as other factors may have influenced the response.

Part B of the questionnaire captured the cost of services provided by asking questions about “amount spent” during the current visit, the service provided, and whether drugs were provided. Knowledge of NHIS and the willingness to enroll were also assessed in this section. Interviewers were trained to rephrase the NHIS questions if the need arose. Each interviewed client was asked about the premium they are willing to pay.

Part C only tested the current capacity of the client to use mobile technology and phone ownership. Clients where asked if they owned a mobile phone and how long they have owned and used a mobile phone. Their ability to send structured text messages using SMS was also assessed in this section of the questionnaire.

Similarly, the health worker’s questionnaire had 3 parts: A, B, and C. Part A asked questions to ascertain the reliability of service in the health facility. They were interviewed about their years of experience. They also responded to how satisfied they were with services provided in the PHC and how satisfied they perceive the community members are with the services provided. This section assessed the staff strength, which is a measure of the human resource capacity in a PHC. It has a direct bearing on the quality of service. Human resource is also a factor of the cost of providing service in a health facility.

Part B assessed the cost of services at the facility. The questions “drugs given,” “cost of antenatal care,” “cost of outpatient department,” and “cost of delivery” were all used to ascertain and document the eventual cost of services in the health facilities. Their awareness and knowledge of NHIS was also assessed in this section. Part C assessed the health worker’s phone ownership and capacity for using phone for SMS and other applications.

Similarly, key informant interview was conducted for a midmanagement HMO representative, an officer-level staff of a mobile network operator, and a mobile money agent.

### Statistical Analysis

The survey data were analyzed using Statistical Package for Social Sciences software (version 22) [26]. Descriptive statistics using the cross-tabulation functionality in most cases were used for the analysis. The analysis considered the study hypothesis that mobile health insurance was both reliable and sustainable and requires adequate cost documentation to demonstrate investment case. The operations of the current PHCs were analyzed and triangulated against the interview data from the clients and health workers. The cost-based price model based on time and material as described by Heizer and Render [27] was used to compare expenditure and income to help arrive at a costing model. The cost incurred by a health facility, private or public, was classified into 4 groups: personnel, fixed equipment, medical consumables, and operating costs. The operating cost was further divided into 4 subsections. But medical consumables were not collected during this study. Further details of the results are discussed in the Results section.

The responses were thematically analyzed in relation to reliability and sustainability indicators [28,29]. The costs of antenatal, delivery, and immunization services were analyzed for variance in mean between public and private primary health care facilities. The variance was also tested for significance using the Student *t*-test at a confidence level of 95%.

## Results

### Demographic Characteristics

All 100-client respondents were expectant or new mothers; 51 (51%) were aged 18-34 years, 48 (48%) were aged 35-49 years, and only 1 (1%) was aged more than 50 years.

Only 25% of clients had visited a health facility in the last month, and 32 (32%) in the last quarter. Fifteen (15%) had visited a health facility within 6 months and 18 (18%) within the last year; 10 (10%) had not visited any health facility in over a year. Twenty-three (23%) of interviewed women had not been sick for over a year, and 26 (26%) were sick between 6 and 12 months.

**Table 1:** Clients’ mean last clinic visit and last period of illness in months by age group.

Age group (years)	Mean last clinic visit (months)	Mean last period of illness (months)	n (%)
18-34	4.12	6.48	51 (51)
35-49	2.61	7.30	48 (48)
≥50	24.00	1.50	1 (1)
Total	3.01	6.34	100 (100)

### Service Rating

**Figure 2:**
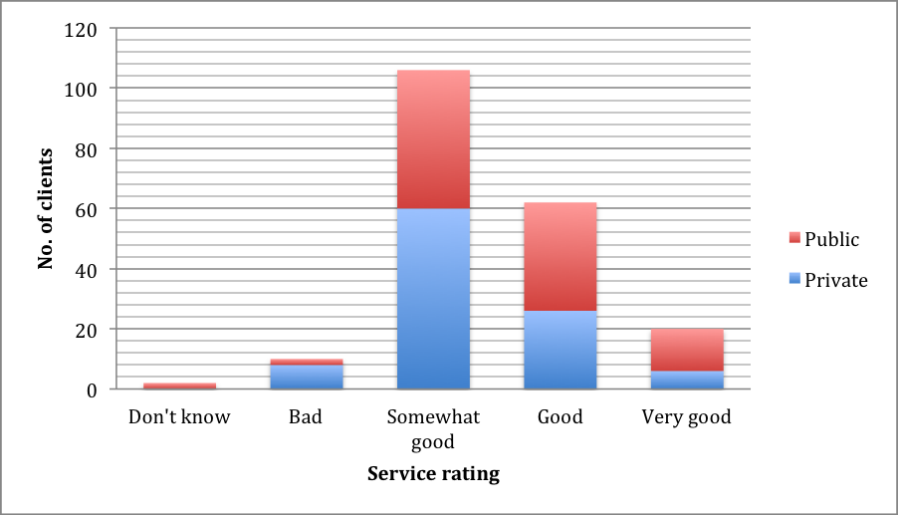
Service rating by clients.

Figure 2 shows moderate variations in service perception rating between respondents in public PHCs and private PHCs: 94% of respondents said the service was between “somewhat-good” and “very good” in public PHCs. This is considered a measure of reliability of the operation, and a mobile app–based health insurance enrolment system could benefit from this perception-based vote of confidence. Although other factors might have influenced this output, the proportion of clients who would recommend the clinics to others was 89%.

The health facility staff strengths were also analyzed, as depicted in Table 2; 53 (53%) of interviewed health facility staff members were female.

**Table 2:** PHC type and staff strength in Abuja (N=100).

PHC^a^type	1-5 Staff n (%)	6-10 Staff n (%)	≥11 Staff n (%)	N (%)
Public PHC	5 (10)	7 (14)	38 (76)	50 (100)
Private PHC	6 (12)	20 (40)	24 (48)	50 (100)
Total	11 (11)	27 (27)	62 (62)	100 (100)

^a^PHC: primary health center.

### Cost Documentation

The monthly expenditures incurred by PHCs were grouped into personnel, operations, fixed equipment, and medical consumables costs. The personnel expenditure primarily included clinical and nonclinical staff time; fixed equipment costs; and covering equipment such as couches, beds, building, and so forth. The operation cost was further subdivided into power, water, paper printing, and transportation costs. The private PHCs are entirely self-funded for all categories of expenditure, whereas their public equivalents have personnel cost completely covered and receive unstructured government subsidy for operations. The level of subsidy depended on various factors such as the proximity of the health facility to the client, client load, and even political interests. In other cases, some of the public health facilities do not receive subsidies.

On the other hand, the health facility income stream was analyzed based on responses from clients and health workers. The results of the analyses are summarized in Table 3. These income streams were categorized by health facility and service type. Table 3 shows a wide variation between out-of-pocket cost of service in public and private PHCs for the 3 services surveyed. The service costs were enquired as a range instead of a discreet amount to address privacy concerns. None of the 100 clinics visited were accepting clients on any form of insurance.

**Table 3:** Service costs as reported by health facility staff interviewed.

PHC ownership		Cost of antenatal service (₦) (*P*<.001)	Cost of OPD^a^consultation (₦) (*P*<.001)	Cost of delivery service (₦) (*P*<.001)
Public				
	n	50	50	50
	Mean	444.5	435.5	426.5
	Standard deviation	541.2231	539.7704	538.1601
Private				
	n	50	50	50
	Mean	2691.44	2008.46	2691.44
	Standard deviation	1472.0359	1538.7633	1472.0359
Total				
	N	100	100	100
	Mean	1567.97	1221.98	1558.97
	Standard deviation	1578.7391	1393.1766	1584.7048

^a^OPD: outpatient department, PHC: primary health center.

The cost of service as reported by the interviewed health workers, shown in Table 3, varied widely between the private PHCs and public PHCs. This difference was consistent across the 3 services assessed.

**Table 4:** Amount spent for health service on clinic visit day.

PHC ownership		Amount spent at clinic today (₦) (*P*<.001)	Premium willing to pay (₦) (*P*>.99)
Public			
	n	50	50
	Mean	1691.42	2380.270
	Standard deviation	1953.861	1736.8265
Private			
	n	50	50
	Mean	4291.04	2380.350
	Standard deviation	2663.122	1427.1472
Total			
	N	100	100
	Mean	2991.23	2380.310
	Standard deviation	2665.777	1581.4980

**Figure 3:**
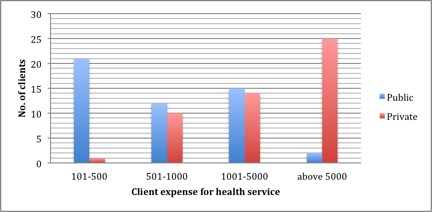
Comparison of private and public service expense cost by health facility.

Similarly, client responses to amount spent for health service as shown in Table 4 also varied widely between private and public PHCs. In contrast, clients in private and public health facilities are willing to pay similar average for family insurance premium as is indicated by *P*>.99. This average amount they are willing to pay is lower than current premium costs. Service costs, particularly for antenatal service, were significantly different in both public and private PHCs as shown by *P*<.001, and any difference cannot be attributed to chance. This difference is clearly demonstrated in Figure 3.

As shown in the figure, the clients interviewed at the public PHCs spent less than those at private PHCs. On the interview day, 21 clients (41%) in public versus 1 (2%) in private PHCs reported spending between ₦ 100 and ₦ 500. The other extreme also shows that half of the clients (n=25; 50%) at private clinics indicated spending more than 5000 on the interview day, whereas only 4 clients (8%) reported doing the same in public clinics.

**Figure 4:**
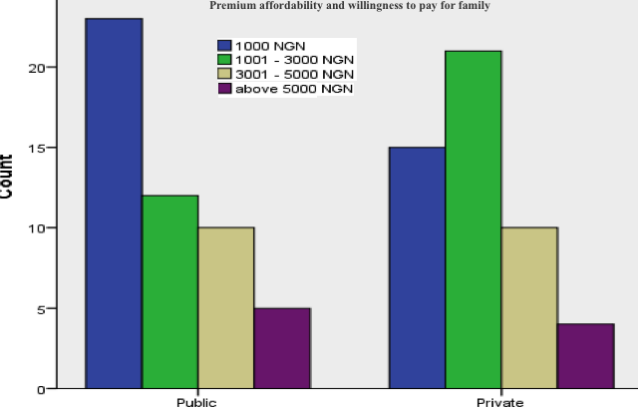
Premium that the interviewed clients are willing to pay per family per annum.

The client interview data for premium affordability and willingness to pay for families were marginally different from health provider interviews, as shown in Figure 4.

**Table 5:** Services provided by PHC^a^type.

Service provided	Private PHC (%)	Public PHC (%)	n (%)
Antenatal attendance	25 (50)	23 (46)	48 (48)
Immunization attendance	12 (24)	24 (48)	36 (36)
Delivery attendance	13 (26)	3 (6)	16 (16)

^a^PHC: primary health center.

Estimates provided in Table 6 are costs documentation averages based on key informant interviews for a fully functional software system. The prevailing inflation and current cost of services were factored in this computation. This, in reality, of course may vary from vendor to vendor and depending on the sophistication of different systems. The 5-year cost expenditure for a fully functional PHC software system was estimated to be $115,425. This was on the assumption that each facility will deploy a cloud computing software system. This can be significantly lower when an LGA for instance pools resources to cover the initial capital investment across board.

**Table 6:** Software setup and operation expenditure.

Year	Seed fund expense	Estimate ($)
Year 1 (Immediate)	One-time software setup	29,800.00
	One-time infrastructure cost and cloud computing setup cost	50,000.00
	Yearly support and maintenance	10,000.00
	Training software administrator	31.25
	Other personnel costs	20,000.00
	Total estimated seed fund for software	109,831.25
		
Year 1	Annual PHC^a^expense	
	Annual data and SMS ($18.72 × 2 × 12)	450.00
	Mobile device (10″ tablet)	562.50
	Spare mobile device	562.50
	Annual device replacement (20% device value)	112.50
	Annual electricity for device charging	300.00
	Training of 5 PHC staff members	156.25
	Total year 1 cost per PHC	2,143.75
Years 2, 3, 4, and 5^b^	Annual data and SMS ($18.72 × 2 × 12)	450.00
	Annual device replacement (20% device value)	112.50
	Annual electricity for device charging	300.00
	Total 1-year recurrent cost per PHC	862.50
	4 Year Recurrent cost	3,450.00
	Total 5-year cost per PHC	115,425.00

^a^PHC: primary health center, SMS: short messaging service.

^b^Yearly recurrent cost.

Excluding drugs and other medical supplies, other annual expenditure expected per PHC for personnel cost and operation costs detailed in Table 7. These estimates were obtained from the prevailing electricity cost and generator fueling costs. Excluding medical consumables does not directly affect the cost of running a digitalized PHC, as it has no effect in this cost-documentation exercise.

**Table 7:** Personnel and operation expenditure of a primary health center^a^.

Serial number	Monthly expenditure	Unit annual rate ($)	Number of personnel	Amount ($)
1	Clinical and nonclinical personnel	3,750	10	37,500
2	Operation costs			1875
	Total			39,375

^a^Excluding medical consumables in a digitized primary health center, as it has no effect in this cost-documentation exercise.

Operational costs summarized in Table 7 can be categorized into system management, transaction fees, hardware and software maintenance, training, and software usage support. The income stream for each PHC can be computed for antenatal and delivery based on their current service rates as received from the interviews. As listed in Table 3, the mean cost of antenatal services is ₦444 ($2.2) for public PHCs and ₦2691 ($13.5) for private PHCs. On the basis of conservative estimates based on key informant interviews with key stakeholders, 50 clients per month per health facility was used to compute the estimated enrolment income. Key informant interviews quoted monthly client load for PHCs as being between 50 and 600 clients, and we assumed that 25 deliveries would be an appropriate estimate per health facility. If the income per health facility is based on the health insurance enrolment for recommended minimum 1000 persons/families. At the proposed estimate for willingness to pay that is at ₦2380 ($11.9) per family per month. The annual premium income from each family will add up to ₦28,560 ($143.5) if they are charged the average amount they are willing to pay (Table 4). This means that for any PHC that has up to 1000 families enrolled for their insurance, the annual premium income will add up to $143,500.

The key informant interview with a midmanagement HMO representative indicates that they reimburse for services using a combination of fee-for-service and capitation within 30 days of claims submission. The 30 days allows for vetting and verification. The most common reason for delayed reimbursement was “missing or incomplete detail.” The main reason for client service denial was attributed to misunderstanding of service level availability for selected plans and list of hospitals including those for referrals. They noted that enrollees complain most about perceived “substandard drugs” given as against out-of-pocket payees. Health facilities are currently reimbursed through bank wire irrespective of location. This HMO indicated that the most basic package for a family of 4 per annum costs ₦90,000 ($452). When enquired if they encourage monthly premium payments, the response was no. On the other hand, an individual will have to enroll with a premium in the range of ₦20,000-450,000 ($100.5-$2261.3), depending on service coverage and hospital selection.

Although a formal interview with a representative of a mobile network operator could not be conducted or because of several reasons, in an informal interview, an officer level staff explained that it is possible to enroll for health insurance using SMS or unstructured supplementary service data and that it is a part of business priority interest for his organization. Similarly, a mobile money agent interviewed to assess the viability of premium payment and service reimbursement to health facilities through mobile money indicated that mobile money business was neither lucrative nor widespread in reach to support the enterprise.

## Discussion

The major findings from this study are that there is wide disparity in cost of payment for health services between private and public PHC institutions in Abuja, Nigeria. The results show that the deployment of a mobile-supported scalable insurance scheme will require significant investment to set up and operate. The interviews indicate that although services are not at an optimal level, clients are generally happy with the service they currently receive. This, we believe, may be related to their experience and exposure. The results of a recent survey conducted by Sambo et al in Kaduna State in north-western Nigeria were consistent with the results of this study on maternal neonatal and child health service costs [30]. However, Sambo et al only focused on public PHCs in their research. Our results are consistent with those of a similar study conducted by Pathfinder International, showing that the use of point-of-care mobile app can significantly improve quality of care in Abuja, Nigeria [31]. In the same study, the Pathfinder International team demonstrated that a significant number of the health workers were happy using the electronic point-of-care tool as against using paper forms. However, the low cost of services at public PHCs has often been associated to the low quality of care provided. This also explains why although the private PHCs charge more than 200% when compared to their public equivalent, the number of deliveries conducted in the private hospitals still remains at almost 4 times of that in public PHCs, as can be seen in Table 4. The effect of introducing mobile health insurance in primary health facilities in Abuja, Nigeria, can be assessed through the implementation of existing health information communication technology, adopting a model similar to the proposed model [31]. The pilot of CommCare point-of-care decision support by Pathfinder International in some facilities in Abuja and Nasarawa, Nigeria, demonstrated that health facility operations were not affected negatively by the introduction of point-of-care technology [13]. We build our premise on this: the introduction of digital job aid is not likely to reduce the reliability of services of the health facilities using it. Other factors are proximity of the PHCs to the client’s residence, irregular supply of drugs and consumables, and so forth. Of the health facility representatives interviewed, 62% indicated having 11 staff members or more, and this was consistent for both private and public PHCs. This however does not always translate to improved quality of care. Visits to these health facilities often show striking contrasts, with not more than 3 staff members at any given time. It was not clear why this is so; however, key informant interviews suggested duty rotation as a possible cause. Another factor worthy of note is that the available data do not distinguish between midwives and community health extension workers, which might be of interest for a future study.

The key informant interview with the HMO showed that the current premium pricing may not be realistic, as only 10% (n=10) of clients interviewed were willing to pay a cumulative of ₦20,000 ($100.5) per family per annum. The HMO reportedly charges this minimum per individual and about ₦100,000 ($502.5) per family, when we consider that many public health facilities may still need a significant initial investment to meet certain service delivery quality standards that will drive demand for health services. The need for alternate and seed funding becomes important for improving both infrastructure and quality level and to subsidize the premium pricing.

In February 2013, a leading Nigerian newspaper, the *National Mirror*, published an article showing that Nigerian telecommunication subscribers spent as much as 1.28 trillion ($6.4 billion) in the year 2012 for voice communications alone [32]. If the current call rates are taxed an additional 0.01 (equivalent of $0.00005), as opposed to the current 0.30 per second for calls within the country, a whopping income of 170 billion ($854 million) will be generated annually. This fund, when properly managed, can be used to fill the gap the seed funds were expected to fill and alleviate the quality concerns in participating PHCs across the country. Furthermore, when quality improves, demand is expected to increase, and as such, insurance alongside its mobile-supported management mechanism can be easily sold. However, this is only an assumption, as it requires political support for effective implementation. Seed funding to improve current PHC infrastructure can also be acquired through donor funding. Others have advocated for sin tax to bridge this gap.

The Nigerian government has provided a minimum of 1% of its consolidated national funds for health care through the National Health Law 2014 [33]. Both NHIS and National Primary Health Care Development agency statutorily receive more than 50% of this fund. This fund can support the infrastructure and lay adequate foundation for a mobile-supported, CBHI and point-of-care service. Funds such as the aforementioned will make health and application of mobile technology to health care both profitable and equitable.

## Limitations

During this study and analysis, the cost of health services at the referral facility (secondary and tertiary health facilities) was assumed to be the same as in the primary health facility and the mean computed from the interviews. However, this is not always so, as the treatment increasingly becomes expensive as we move from primary care to secondary to tertiary. The income analyses were conducted using out-of-pocket payment extrapolation. The actual proposed income profile will vary slightly based on insurance enrolment and buy-in by each community. According to Abuja FCT, millennium development goals office, drug supplies to these public health facilities have often been inadequate [34]. This is often worsened by the poor commodity logistics at this level of care in the country. Investigations through informal discussions show that these hitherto insufficient drugs are sometimes wasted because of poor logistics. The health facility staff then makes up for these shortfalls by adopting a widely known method locally called “drug revolving.” In this method, they are allowed to buy drugs to augment supplies from the government and use generated funds to maintain drug supply in the health facility; however, they are not expected to make profits. Inconsistency in supply of these drugs, which sometimes do not reach the health facilities, and other equipment and structural variations makes it pertinent for treatment price to vary from health facility to health facility. This variation can even be wider between public and private PHCs. This poses a huge challenge for uniform pricing and particularly for premium determination. This is critical as NHIS is emphasizing its social insurance role and angling towards mandatory health insurance with the states as drivers. Moreover, statistics available show that private practitioners bear the burden of a larger percentage of the population in Abuja, Nigeria, at this level of care under consideration in FCT, as they own 68% (380 of 559) of the PHCs [4]. The assumption that a seed fund can help fill the identified gap also recognizes that there are other sectors (Agriculture, Education, Environment, Power etc) competing with health for priority investment and will have to harmonize asks for effectiveness.

## Conclusions

Mobile health insurance presents an opportunity for wider expansion of insurance adopters. It was easy to establish that a mobile enrolment system will improve efficiency and reliable. The interests demonstrated by mobile network operators and health management organizations demonstrate business interest and willingness to participate. However, profitable cost for identified stakeholders would hover around the private PHCs’ mean service costs, still requiring significant subsidy. This means that to achieve universal health coverage, insurance costing model must consider identified variations. This study successfully demonstrated that mobile supported enrolment, encounter management, treatment verification, and claims management and reimbursements can be efficient and sustainable. It also shows that using technology can aid in accountability and thus reliability of CBHI financing and reimbursement. This study successfully documented income and expenditure associated with personnel, fixed equipment, and operation, as they influence the adoption of mobile insurance-management system. Costs for medical consumables have been a topic of many other researches and were not considered in this study.

## Abbreviations

CBHI: community-based health insurance
FCT: federal capital territory
GSM: Global System for Mobile Communications
HMO: health maintenance organization
LGA: local government area
NHIS: National Health Insurance Scheme
PHC: primary health center

## References

[1] Marwa B, Njau B, Kessy J, Mushi D (2013). Feasibility of introducing compulsory community health fund in low resource countries: views from the communities in Liwale district of Tanzania. BMC Health Serv Res.

[2] World Bank (2010). World DataBank: Developement Indicators.

[3] Okonofua F, Lambo E, Okeibunor J, Agholor K (2011). Advocacy for free maternal and child health care in Nigeria--Results and outcomes. Health Policy.

[4] FMoH (2012). A Directory of Health Facilities in Nigeria - 2011.

[5] NHIS (2013). "No T".

[6] Rao KD, Waters H, Steinhardt L, Alam S, Hansen P, Naeem AJ (2009). An experiment with community health funds in Afghanistan. Health Policy Plan.

[7] Roy P (2013). Community Based Health Insurance Schemes And Social Work Implications For Health Insurance Practices For The Poor. Indian Streams Research Journal.

[8] Drossos A (2002). http://www.alexdrossos.ca/downloads/ffscapitation.pdf.

[9] Slattery E, Clancy KX, Harewood GC, Murray FE, Patchett S (2013). Does the cost of care differ for patients with fee-for-service vs. capitation of payment? A case-control study in gastroenterology. Ir J Med Sci.

[10] Cox T (2011). Exposing the true risks of capitation financed healthcare. J Healthc Risk Manag.

[11] Frakt AB, Mayes R (2012). Beyond capitation: how new payment experiments seek to find the 'sweet spot' in amount of risk providers and payers bear. Health Aff (Millwood).

[12] Dixon J, Tenkorang EY, Luginaah I (2013). Ghana's National Health Insurance Scheme: a national level investigation of members' perceptions of service provision. BMC Int Health Hum Rights.

[13] Onwujekwe O, Onoka C, Uguru N, Nnenna T, Uzochukwu B, Eze S, Kirigia J, Petu A (2010). Preferences for benefit packages for community-based health insurance: an exploratory study in Nigeria. BMC Health Serv Res.

[14] DeRenzi BL, Findlater L, Payne J, Birnbaum B, Mangilima J, Parikh T, Borriello G, Lesh N (2012). Improving community health worker performance through automated SMS.

[15] NPHCDA Minimum standards for primary health care in Nigeria.

[16] Smith KV, Sulzbach S (2008). Community-based health insurance and access to maternal health services: evidence from three West African countries. Soc Sci Med.

[17] Uzochukwu B (2010). Implementing community based health insurance in Anambra state, Nigeria.

[18] NCC Telecoms subscribers in growth per quarter.

[19] Aranda-Jan CB, Mohutsiwa-Dibe N, Loukanova S (2014). Systematic review on what works, what does not work and why of implementation of mobile health (mHealth) projects in Africa. BMC Public Health.

[20] Odigie VI, Yusufu LM, Dawotola DA, Ejagwulu F, Abur P, Mai A, Ukwenya Y, Garba ES, Rotibi BB, Odigie EC (2012). The mobile phone as a tool in improving cancer care in Nigeria. Psychooncology.

[21] Tomlinson M, Rotheram-Borus MJ, Swartz L, Tsai AC (2013). Scaling up mHealth: where is the evidence?. PLoS Med.

[22] Kumar S, Bauer K (2009). The business case for implementing electronic health records in primary care settings in the United States. J Revenue Pricing Manag.

[23] Global Health Workforce Nigeria launches 'Saving One Million Lives' by 2015 initiative.

[24] mHealth Alliance (2013). Baseline Evaluation of the mHealth Ecosystem and the Performance of the mHealth Alliance.

[25] Foh K (2011). Mobile health in the health insurance industry.

[26] IBM (2014). SPSS for Statistics v.22.

[27] Heizer J, Render B (2009). Operations Management, Flexible Version, 9th Edition.

[28] Nidumolu R, Prahalad C, Rangaswami M (2013). Why sustainability is now the key driver of innovation. IEEE Eng Manag Rev.

[29] mHealth Alliance, Vital Wave Consulting (2013). Sustainable Financing for Mobile Health (mHealth): Options and opportunities for mHealth financial models in low- and middle-income countries.

[30] Sambo MN, Abdulrazaq GA, Shamang AF, Ibrahim AA (2013). Household cost of antenatal care and delivery services in a rural community of Kaduna state, northwestern Nigeria. Niger Med J.

[31] Mcnabb M, Chukwu E, Ojo O, Shekhar N, Gill C, Salami H, Jega F (2014). Assessment of the Quality of Antenatal Care Services Provided by Health Workers using a Mobile Phone Decision Support Application in Northern Nigeria: A Pre / Post-intervention Study. PLOS one.

[32] Azeez K (2013). National mirror.

[33] Federal Ministry of Health (2014). National Health Law. Nigeria: National Assembly.

[34] FCT MDG (2015). Health Services.

